# Ferroptosis in Oral Cancer: Mechanistic Insights and Clinical Prospects

**DOI:** 10.3390/cells14211685

**Published:** 2025-10-27

**Authors:** Jaewang Lee, Jong-Lyel Roh

**Affiliations:** 1 Department of Otorhinolaryngology-Head and Neck Surgery, CHA Bundang Medical Center, CHA University, Seongnam 13488, Republic of Korea; 2 Logsynk, Seoul 06153, Republic of Korea; 3 Department of Biomedical Science, General Graduate School, CHA University, Pocheon 11160, Republic of Korea

**Keywords:** ferroptosis, oral cancer, therapy resistance, biomarkers, nanomedicine

## Abstract

**Highlights:**

**What are the main findings?**

1. Ferroptosis represents a targetable vulnerability in oral squamous cell carcinoma (OSCC).
2. Genetic, epigenetic, and metabolic regulators, including *GPX4*, *SLC7A11*, *NFE2L2*, and *ACSL4*, shape ferroptosis sensitivity.
3. Repurposed drugs, natural compounds, and nanomedicine formulations effectively induce ferroptosis in OSCC.

**What are the implications of the main findings?**

4. Ferroptosis activation enhances the therapeutic efficacy of cisplatin, radiotherapy, and immunotherapy in OSCC.
5. Ferroptosis-related biomarkers and non-cancer oral disease mechanisms broaden the clinical and translational relevance of ferroptosis.

**Abstract:**

Ferroptosis, an iron-dependent form of regulated cell death characterized by lipid peroxidation, has emerged as a pivotal vulnerability in oral squamous cell carcinoma (OSCC). This review provides an overview of ferroptosis mechanisms and their implications for OSCC pathobiology and therapy. OSCC cells exhibit heightened reliance on anti-ferroptotic defenses such as GPX4, SLC7A11, FSP1, and Nrf2, and disrupting these pathways suppresses tumor growth and restores sensitivity to chemotherapy, radiotherapy, and immunotherapy. Genetic and epigenetic regulators, including p53, PER1, circ_0000140, and STARD4-AS1, critically modulate ferroptotic sensitivity, while metabolic enzymes such as ACSL4, LPCAT3, and TPI1 link ferroptosis to cellular plasticity and resistance. Preclinical studies highlight the promise of small-molecule inhibitors, repurposed agents (e.g., sorafenib, artesunate, trifluoperazine), natural compounds (e.g., piperlongumine, Evodia lepta, quercetin), and nanomedicine platforms for targeted ferroptosis induction. We further address ferroptosis within the tumor microenvironment, highlighting its immunogenic and context-dependent dual roles, and summarize genomic and transcriptomic evidence linking ferroptosis-related genes to patient prognosis. Beyond cancer, ferroptosis also contributes to non-malignant oral diseases, including pulpitis, periodontitis, and infection-associated inflammation, where inhibitors may protect tissues. Despite these advances, clinical translation is constrained by the lack of safe ferroptosis inducers and validated biomarkers. Future research should focus on developing pharmacologically viable GPX4 inhibitors, refining biomarker-driven patient stratification, and designing multimodal regimens that combine ferroptosis induction with standard therapies while preserving immune and tissue integrity. Ferroptosis therefore represents both a mechanistic framework and a translational opportunity to reshape oral oncology and broader oral disease management.

## 1. Introduction

Oral squamous cell carcinoma (OSCC), the predominant histological subtype of oral cancer, accounts for more than 90% of all oral malignancies and represents a major global health challenge [1]. Epidemiologically, tobacco and alcohol remain the dominant risk factors, while human papillomavirus (HPV) infection has emerged as an additional etiological factor in specific populations [2]. Indeed, a recent Global Burden of Disease (GBD) analysis reported over half a million new cases and more than 300,000 deaths from lip, oral, and pharyngeal cancers worldwide in 2019, with striking geographic disparities; the highest mortality was observed in low- and middle-sociodemographic index (SDI) regions, largely attributable to tobacco and alcohol use [3]. Despite improvements in surgical techniques, radiotherapy, and chemotherapy, the 5-year survival rate for patients with OSCC has stagnated around 50–60%, primarily due to high recurrence rates, therapeutic resistance, and the presence of aggressive disease at diagnosis [4,5]. The clinical management of OSCC is further complicated by molecular heterogeneity, epithelial–mesenchymal transition (EMT), cancer stem cell (CSC) plasticity, and an immunosuppressive tumor microenvironment (TME), all of which contribute to treatment resistance and poor outcomes [6,7]. Tumor cells frequently acquire resistance to apoptosis through multiple mechanisms, including *TP53* mutations, overexpression of anti-apoptotic BCL-2 family proteins, caspase inactivation, and metabolic rewiring, that enhance redox homeostasis. Moreover, EMT and CSC enrichment further reduce apoptotic susceptibility, leading to residual disease and recurrence. Consequently, apoptosis-based therapeutic paradigms have proven insufficient to eradicate resistant OSCC subpopulations, underscoring the need for alternative regulated cell death (RCD) pathways such as ferroptosis [8].

In this context, ferroptosis has emerged as a promising avenue of investigation. Ferroptosis is a regulated form of cell death that is mechanistically and morphologically distinct from apoptosis and autophagy-dependent cell death (ADCD) [9]. It is driven not by generalized oxidative stress, but by iron-dependent lipid peroxidation of polyunsaturated phospholipids, which compromises membrane integrity once the antioxidant defenses fail [9,10]. The process is initiated when the antioxidant defense system, particularly the glutathione (GSH)–glutathione peroxidase 4 (GPX4) axis, fails to neutralize the accumulation of polyunsaturated fatty acid (PUFA)-containing phospholipid (PL) hydroperoxides [11,12]. According to the Nomenclature Committee on Cell Death, ferroptosis is thus classified as a distinct subtype of RCD, separate from accidental cell death forms such as necrosis [13]. Morphologically, ferroptosis features distinctive mitochondrial changes, including reduced or absent cristae and condensed outer membranes, without the nuclear fragmentation typical of apoptosis [14,15]. The discovery of ferroptosis has opened new opportunities to exploit metabolic vulnerabilities in cancer cells, particularly those that have acquired resistance to conventional therapies [16].

OSCC cells exhibit several hallmarks that may render them especially vulnerable to ferroptosis [17,18]. First, the phenomenon of “iron addiction” in rapidly proliferating malignant cells results in the elevated uptake of iron through transferrin receptor 1 (TfR1) and dysregulated ferritin metabolism, creating a labile iron pool (LIP) that drives oxidative stress [19]. Second, OSCC cells display enriched PUFA-PL, particularly through the action of acyl-CoA synthetase long-chain family member 4 (ACSL4) and lysophosphatidylcholine acyltransferase 3 (LPCAT3), which facilitate the incorporation of arachidonic acid and adrenic acid into membrane phosphatidylethanolamines (PEs)—a key step in ferroptotic lipid peroxidation [20,21]. Third, metabolic reprogramming and antioxidant upregulation, including enhanced expression of GPX4, nuclear factor erythroid 2-related factor 2 (Nrf2), and ferroptosis suppressor protein 1 (FSP1), enable OSCC cells to escape oxidative stress while simultaneously generating a “ferroptosis vulnerability” when these pathways are inhibited [22,23].

Emerging preclinical studies strongly support the therapeutic potential of ferroptosis induction in OSCC. For instance, inhibition of GPX4 or system Xc^−^ (SLC7A11/SLC3A2), as well as pharmacological ferroptosis inducers such as erastin and RSL3, have been shown to trigger cell death in OSCC cell lines (CAL27, SCC9, HSC3) and to enhance sensitivity to chemotherapy [24,25]. Recent investigations into cisplatin-resistant OSCC revealed that resistant cells maintain lower basal lipid peroxidation and reactive oxygen species (ROS) compared to sensitive counterparts, implicating ferroptosis resistance as a central mechanism of chemoresistance [26]. Conversely, agents that suppress GPX4, modulate iron homeostasis, or disrupt antioxidant defenses can re-sensitize resistant OSCC cells to cisplatin, highlighting ferroptosis as a leverage point in overcoming treatment failure [8,27].

Beyond chemotherapy resistance, ferroptosis also intersects with several hallmarks of OSCC biology. The period circadian regulator PER1, for example, promotes ferroptosis by downregulating hypoxia-inducible factor-1α (HIF-1α), thereby increasing ROS and lipid peroxidation; loss of PER1 expression suppresses ferroptosis and accelerates tumor progression [24]. Similarly, oncogenic pathways involving p53 mutations, long non-coding RNAs (lncRNAs) such as MALAT1 and STARD4-AS1, and circular RNAs (circRNAs) such as circ_0000140, influence ferroptosis regulation and drug resistance [28,29,30]. In addition, metabolic enzymes such as triosephosphate isomerase 1 (TPI1) and palmitoyl-protein thioesterase 1 (PPT1) have been shown to modulate ferroptosis sensitivity in OSCC cells, further underscoring the intimate link between ferroptosis and the metabolic adaptability of oral cancer [31,32].

Natural products and bioactive compounds have also attracted attention as ferroptosis inducers in oral cancer. Piperlongumine, a natural alkaloid, has been shown to induce ferroptosis in OSCC cells by suppressing SLC7A11 and GPX4 while promoting ROS accumulation; its activity is further enhanced in combination with the glutaminase inhibitor CB-839 [33]. *Evodia lepta* extract selectively induces ferroptosis in tongue squamous cell carcinoma cells through *GPX4* and *HSPA5* downregulation, while simultaneously reducing PD-L1 expression, suggesting both cytotoxic and immunotherapeutic potential [34]. These findings highlight the possibility of integrating ferroptosis-based therapies with immunomodulation, an area of great relevance given the limited efficacy of current immune checkpoint inhibitors in OSCC.

Technological advances in nanomedicine further expand the therapeutic landscape. Nanoparticles designed for ROS amplification, GSH depletion, or photothermal responses have demonstrated synergistic ferroptosis induction in OSCC models, offering a novel means of targeted drug delivery and minimizing systemic toxicity [35]. Such platforms exemplify how ferroptosis modulation can be integrated into combination treatment strategies that exploit metabolic stress, immune activation, and conventional cytotoxic therapies.

Importantly, ferroptosis does not act in isolation, but interacts with other RCD pathways, including apoptosis, pyroptosis, and autophagy [8]. In OSCC, the interplay between ferroptosis and autophagy is particularly noteworthy, as ferritinophagy-mediated iron release contributes to ferroptotic cell death, and combined induction of autophagy and ferroptosis has been proposed as a synergistic therapeutic strategy [36]. This crosstalk expands the potential to harness multiple death modalities to overcome the redundancy and adaptability of cancer survival mechanisms.

Taken together, accumulating evidence positions ferroptosis as a critical vulnerability in OSCC biology and treatment. Its distinct mechanism—rooted in iron metabolism, lipid peroxidation, and redox imbalance—offers unique opportunities to overcome conventional therapy resistance and exploit metabolic dependencies in oral cancer. However, major challenges remain, including the need to identify reliable biomarkers of ferroptosis sensitivity, to mitigate potential off-target effects, and to translate preclinical findings into clinical trials. The aim of this review is therefore to provide a comprehensive synthesis of current knowledge on ferroptosis in OSCC, spanning mechanistic insights, molecular regulators, therapeutic strategies, and clinical implications. By integrating emerging evidence from molecular biology, pharmacology, and translational research, we seek to highlight ferroptosis as both a conceptual and practical framework for developing next-generation therapies against oral cancer. This review provides an updated and mechanistically focused synthesis of ferroptosis in OSCC, integrating recent molecular insights with translational advances. Specifically, it highlights lipid metabolism–driven ferroptosis regulation, ferroptosis suppressor networks (GPX4–FSP1–DHODH–Nrf2), and emerging therapeutic approaches such as nanomedicine delivery and ferroptosis–immunotherapy combinations. These aspects distinguish the present review from previous broad overviews and underscore its novelty in connecting molecular ferroptosis biology with clinical applications in oral oncology.

## 2. Molecular Basis of Ferroptosis

**Figure 1:**
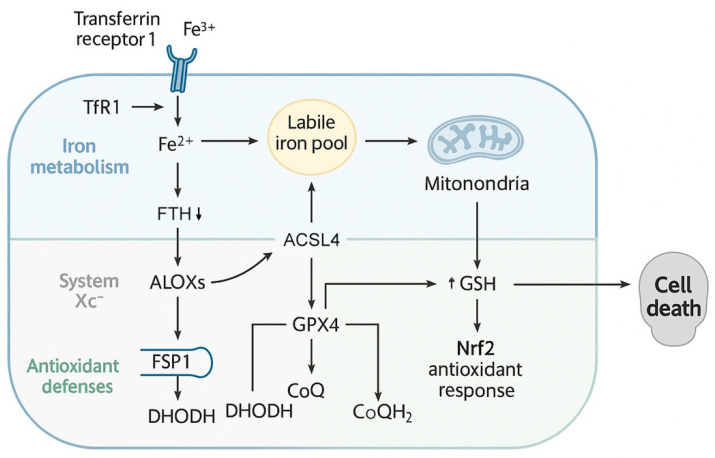
Molecular basis of ferroptosis. Iron uptake via TfR1 and release through NCOA4-mediated ferritinophagy expand the labile iron pool, fueling Fenton chemistry and reactive oxygen species generation. PUFAs are activated by ACSL4, esterified by LPCAT3, and oxidized by lipoxygenases (ALOXs) or POR, leading to lethal lipid peroxidation. System Xc^−^, composed of SLC7A11 and SLC3A2, imports cystine for GSH synthesis, maintaining redox homeostasis through the GSH–GPX4 axis. Inhibition or depletion of GSH disrupts this antioxidant defense, resulting in lipid peroxide accumulation and ferroptotic cell death. This process is antagonized by the system Xc^−^ antiporter, glutathione (GSH), and GPX4, with parallel protection provided by FSP1, DHODH, and the Nrf2 transcriptional program. Abbreviations: ACSL4, acyl-CoA synthetase long-chain family member 4; ALOXs, arachidonate lipoxygenases; CoQ, coenzyme Q10; CoQH_2_, ubiquinol; DHODH, dihydroorotate dehydrogenase; FSP1, ferroptosis suppressor protein 1; GPX4, glutathione peroxidase 4; GSH, glutathione; LPCAT3, lysophosphatidylcholine acyltransferase 3; NCOA4, nuclear receptor coactivator 4; Nrf2, nuclear factor erythroid 2–related factor 2; POR, cytochrome P450 oxidoreductase; PUFA, polyunsaturated fatty acid; SLC3A2, solute carrier family 3 member 2; SLC7A11, solute carrier family 7 member 11; System Xc^−^, cystine/glutamate antiporter; TfR1, transferrin receptor 1.

Ferroptosis is a unique form of RCD defined by the iron-dependent accumulation of lipid peroxides, and its core biochemical framework integrates multiple layers of cellular metabolism, including iron handling, lipid remodeling, and antioxidant defense (Figure 1) [14]. Understanding these molecular foundations is essential for explaining why certain cancers, such as OSCC, display marked sensitivity to ferroptosis induction [20,37,38]. This section outlines the essential processes that orchestrate ferroptosis, with emphasis on iron metabolism, lipid peroxidation, amino acid and GSH pathways, and additional regulators that fine-tune the ferroptotic response.

### 2.1. Iron Metabolism and the Labile Iron Pool

Iron homeostasis plays a central role in the initiation of ferroptosis. Physiologically, iron is required for DNA synthesis, mitochondrial respiration, and enzymatic reactions. However, excessive intracellular ferrous iron (Fe^2+^) becomes a double-edged sword, as it catalyzes Fenton chemistry, generating hydroxyl radicals that trigger oxidative injury [39]. Iron enters the cell mainly through transferrin receptor 1 (TfR1)-mediated endocytosis of transferrin-bound ferric iron (Fe^3+^). Within endosomes, Fe^3+^ is reduced to Fe^2+^ by six-transmembrane epithelial antigen of the prostate 3 (*STEAP3*) and subsequently released into the cytoplasm via divalent metal transporter 1 (*DMT1*), contributing to the so-called LIP [40,41].

The LIP serves as the catalytic hub of ferroptosis. Elevated Fe^2+^ levels fuel the Fenton reaction, producing hydroxyl radicals that oxidize nearby lipids and propagate lipid peroxidation chains [42]. Under normal conditions, cells buffer excess iron through storage in ferritin, a heteropolymeric protein composed of ferritin light (*FTL*) and heavy (*FTH1*) chains. However, selective autophagic degradation of ferritin, termed ferritinophagy, releases stored iron and amplifies susceptibility to ferroptosis [43]. This process is orchestrated by nuclear receptor coactivator 4 (NCOA4), which shuttles ferritin to the autophagosome, thereby increasing cytosolic Fe^2+^ [44].

Dysregulated ferritinophagy has been implicated in OSCC, where increased iron release enhances ROS generation and sensitizes malignant cells to ferroptosis inducers [45]. Conversely, iron export via ferroportin (*SLC40A1*) and sequestration in mitochondrial ferritin (*FTMT*) can protect cells from ferroptosis [46]. The balance between iron import, storage, and release, therefore, dictates ferroptotic potential. Importantly, many OSCC cell lines demonstrate heightened TfR1 expression and decreased ferroportin activity, reflecting their “iron addiction” and establishing an intrinsic vulnerability to ferroptosis [39,42].

### 2.2. Lipid Peroxidation: The Execution Step of Ferroptosis

The defining event of ferroptosis is the accumulation of oxidized phospholipids, particularly PUFA-PEs [47]. PUFAs, such as arachidonic acid (AA) and adrenic acid (AdA), contain bis-allylic hydrogen atoms that are especially prone to radical attack, making them prime substrates for peroxidation [48]. For these fatty acids to participate in ferroptosis, they must be esterified into membrane phospholipids. This is achieved through the sequential actions of ACSL4, which activates free PUFAs into acyl-CoA intermediates, and LPCAT3, which incorporates them into PL backbones [49].

Once embedded in membranes, PUFA-PEs undergo oxidation via both enzymatic and non-enzymatic pathways. Non-enzymatic lipid peroxidation is largely driven by Fenton-generated hydroxyl radicals, which abstract hydrogen atoms and propagate lipid radical chain reactions. Enzymatic lipid peroxidation involves lipoxygenases (ALOX5, ALOX12, ALOX15) and cytochrome P450 oxidoreductase (POR), which selectively oxygenate PUFA-PEs to form hydroperoxides [50,51]. The PE-binding protein 1 (PEBP1) further facilitates this process by presenting PUFA-PE substrates to ALOX15, amplifying ferroptotic signaling [52].

In OSCC, enhanced expression of ACSL4 has been correlated with aggressive disease phenotypes, while inhibition of ACSL4 diminishes ferroptotic susceptibility [53,54]. Conversely, enzymes that degrade oxidized fatty acids, such as calcium-independent phospholipase A2β (iPLA2β), confer resistance by removing damaged PUFA chains from phospholipids [48]. This dynamic remodeling of lipid composition underlies the heterogeneity of ferroptosis sensitivity across oral tumors.

### 2.3. Amino Acid Metabolism and the System Xc^–^/GSH/GPX4 Axis

Antioxidant defense mechanisms counteract lipid peroxidation and define the threshold for ferroptotic death. Central to this process is the cystine/glutamate antiporter, known as system Xc^−^, which imports cystine in exchange for glutamate [55]. Cystine is rapidly reduced to cysteine, the rate-limiting precursor for GSH synthesis. GSH, in turn, serves as the indispensable cofactor for GPX4, the only enzyme capable of reducing phospholipid hydroperoxides (PLOOHs) into non-toxic alcohols [56].

Inhibition of system Xc^−^ by erastin or sulfasalazine, or direct inhibition of GPX4 by RSL3, eliminates this protective shield, leading to unchecked lipid peroxidation and ferroptosis [9,57]. OSCC cells frequently upregulate SLC7A11, the catalytic subunit of system Xc^−^, as part of an adaptive strategy to neutralize oxidative stress [58]. This overreliance creates a targetable vulnerability; silencing SLC7A11 sensitizes OSCC cells to ferroptosis and impairs tumor growth [59,60,61]. Alternative routes for cysteine supply also influence ferroptosis. The trans-sulfuration pathway converts methionine to cysteine, thereby bypassing system Xc^−^ inhibition. This metabolic plasticity contributes to resistance in some tumors, although its role in OSCC remains under investigation.

### 2.4. Additional Ferroptosis Regulators

FSP1, formerly known as AIFM2, functions as a parallel antioxidant system independent of GPX4. It regenerates reduced coenzyme Q_10_ (CoQ_10_H_2_) using NADPH, which then intercepts lipid radicals and prevents propagation of lipid peroxidation [62,63]. In OSCC, FSP1 upregulation has been linked with therapy resistance, while dual inhibition of GPX4 and FSP1 markedly enhances ferroptosis induction. DHODH, located at the inner mitochondrial membrane, couples pyrimidine synthesis with electron transport by reducing CoQ_10_. Recent studies indicate that DHODH limits mitochondrial lipid peroxidation and serves as a backup system when GPX4 activity is compromised [64]. Inhibiting DHODH may therefore provide a strategy to overcome mitochondrial ferroptosis resistance in OSCC.

Nrf2 is a master regulator of redox balance, activating transcription of antioxidant genes, including SLC7A11, GPX4, and heme oxygenase-1 (HO-1) [65]. Although Nrf2 activation can transiently promote ferroptosis through HO-1-mediated iron release, it generally acts as a potent suppressor of ferroptosis [66]. In OSCC, hyperactivation of Nrf2 correlates with drug resistance, emphasizing its role as a therapeutic barrier [67]. The tumor suppressor p53 exerts dual effects on ferroptosis. On one hand, it represses SLC7A11 transcription, thereby limiting cystine uptake and sensitizing cells to ferroptosis [68]. On the other hand, p53 also enhances antioxidant defenses in certain contexts, delaying ferroptosis onset [69]. In OSCC, p53 mutations disrupt this balance, often promoting ferroptosis resistance [28,70]. Chaperones such as HSPB1 and HSPA5 mitigate ferroptosis by regulating iron uptake and stabilizing GPX4, respectively [71,72]. OSCC studies have reported HSPA5 upregulation as part of an adaptive stress response, reinforcing the link between proteostasis and ferroptosis resistance [34].

Although ferroptosis is mechanistically distinct, it interacts with other RCD modalities. Autophagy contributes by promoting ferritinophagy, thereby releasing iron to fuel ferroptosis [43,73]. Apoptosis and ferroptosis can co-exist, with some compounds inducing mixed features depending on dose and context [74]. In OSCC, combined induction of autophagy and ferroptosis (e.g., with RSL3 and autophagy activator LYN-1604) synergistically suppresses tumor growth [36]. Crosstalk with pyroptosis and necroptosis further complicates the landscape but also provides combinatorial opportunities to target resistant cancer cells [8].

Ferroptosis is distinguished from other cell death modes by its ultrastructural and biochemical hallmarks. Mitochondria undergo profound shrinkage, loss of cristae, and increased membrane density, but nuclei remain morphologically intact without chromatin condensation. At the biochemical level, cells accumulate lipid hydroperoxides, malondialdehyde (MDA), and 4-hydroxynonenal (4-HNE), serving as surrogate markers of ferroptosis [9,75]. In OSCC models, these hallmarks have been consistently observed in response to ferroptosis inducers, validating their utility as diagnostic features in experimental systems [8,76].

In summary, ferroptosis arises from the intersection of iron overload, PUFA peroxidation, and insufficient antioxidant defense. The balance between pro-ferroptotic drivers (iron uptake, ACSL4-mediated lipid remodeling, ferritinophagy) and anti-ferroptotic systems (SLC7A11, GPX4, FSP1, DHODH, Nrf2) determines cellular susceptibility. OSCC cells, by virtue of their iron dependency, PUFA-rich membranes, and altered antioxidant responses, exhibit a unique predisposition to ferroptosis, laying the groundwork for therapeutic exploitation in subsequent sections.

## 3. Ferroptosis in Oral Cancer: Preclinical Evidence

**Figure 2:**
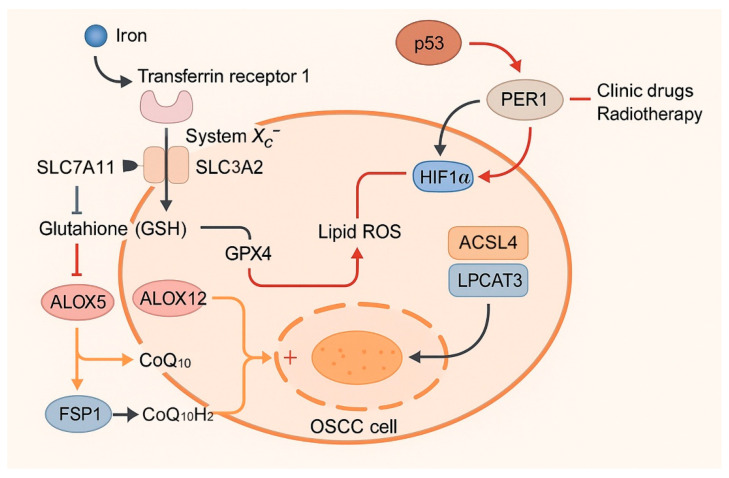
Regulatory landscape of ferroptosis in OSCC. Schematic illustration of selected regulators of ferroptosis in oral squamous cell carcinoma (OSCC). Tumor suppressors such as p53 and PER1 promote ferroptosis by repressing SLC7A11 or lowering HIF-1α, thereby enhancing reactive oxygen species (ROS) and lipid peroxidation. In contrast, oncogenic regulators including AEBP1, PPT1, and CK19 inhibit ferroptosis by activating MAPK signaling, stabilizing GPX4, or suppressing ACSL4 activity. Non-coding RNAs such as circ_0000140 and STARD4-AS1 reinforce ferroptosis resistance, while metabolic enzymes including TPI1 suppress lipid ROS and ACSL4/LPCAT3 enhance ferroptotic sensitivity. Together, these representative and additional regulators generate a ferroptosis-resistant phenotype in OSCC, particularly in drug-resistant and mesenchymal-like cells.

Ferroptosis has emerged as a key vulnerability in OSCC, the most common malignancy of the oral cavity [17]. Preclinical studies using established cell lines, animal models, and bioinformatic datasets consistently indicate that ferroptosis is suppressed in untreated OSCC, and that targeted reactivation of ferroptotic pathways impairs tumor growth, reverses drug resistance, and enhances therapeutic efficacy (Figure 2 and Table 1). This section reviews the major preclinical evidence linking ferroptosis to oral cancer biology, with emphasis on basal suppression of ferroptosis, genetic and epigenetic regulators, metabolic enzymes, chemoresistance, tumor microenvironmental effects, and therapeutic approaches involving natural products and nanomedicine.

### 3.1. Suppression of Ferroptosis in Baseline OSCC Models

A consistent finding across OSCC models is the suppression of ferroptosis at baseline. Established cell lines such as CAL27, SCC9, SCC15, SCC25, HSC2, HSC3, and SAS demonstrate elevated expression of GPX4 and the cystine/glutamate antiporter subunit SLC7A11, which prevent the accumulation of lipid peroxides and dampen ferroptotic sensitivity [24,25,77]. Knockdown of these protective factors or pharmacological inhibition using erastin (system Xc^−^ inhibitor) or RSL3 (GPX4 inhibitor) readily restores ferroptosis in these models, highlighting their critical role in ferroptosis evasion [9,91]. In addition to SLC7A11, system Xc^−^ also requires SLC3A2, and recent evidence shows that reduced SLC3A2 expression in OSCC facilitates immune evasion and metastasis, while its overexpression enhances T cell function and may counteract ferroptosis resistance [78].

Bioinformatic analyses further corroborate these experimental findings. Ferroptosis-related gene (FRG) signatures derived from The Cancer Genome Atlas (TCGA) and Gene Expression Omnibus (GEO) datasets reveal differential expression patterns between OSCC tumors and normal oral epithelium, with high-risk FRGs predicting poor survival and low ferroptosis activity [92,93]. A ferroptosis score (FPscore) developed from transcriptomic profiles correlates with tumor purity, immune cell infiltration, and patient prognosis; patients with high FPscore show longer survival and enhanced responses to immunotherapy, suggesting that ferroptosis status strongly influences the tumor immune microenvironment [94,95].

Several prognostic models based on FRGs have been validated. One study identified *ATG5*, *AKR1C3*, *ALOX15*, and *ACO1* as risk factors, while *MAP1LC3A*, *SCO2*, and *MAP3K5* were protective, and the final three-gene model (ATG5, MAP3K5, MAP1LC3A) predicted overall survival more accurately than clinical features alone [92]. Another model integrating 321 ferroptosis-associated genes with weighted gene co-expression network analysis (WGCNA) narrowed the set to seven genes, generating a prognostic signature with area under the curve (AUC) values of 0.565–0.733 for 1-, 3-, and 5-year survival predictions [76]. Collectively, these findings establish that ferroptosis is suppressed in OSCC at baseline, and its activation may serve as both a prognostic marker and a therapeutic strategy.

### 3.2. Tumor Suppressors and Oncogenes Regulating Ferroptosis in OSCC

Several tumor suppressors and oncogenes are intimately involved in ferroptosis regulation in OSCC. The tumor suppressor p53 enhances ferroptotic sensitivity by repressing SLC7A11 transcription and limiting cystine uptake; however, p53 mutations common in OSCC disrupt this regulation, leading to sustained glutathione synthesis and ferroptosis resistance [68,77]. Restoration of p53 activity, or direct targeting of SLC7A11, re-sensitizes OSCC cells to ferroptosis inducers [82]. In contrast, Nrf2 acts as a master antioxidant regulator that counteracts ferroptosis by transcriptionally activating GPX4, SLC7A11, and HO-1, thus enhancing cellular defense against lipid peroxidation and contributing to therapy resistance in OSCC [66,80]. In addition, *NFE2L1* was recently shown to suppress ferroptosis in OSCC by transcriptionally upregulating HJURP, which stabilizes GPX4 and SLC7A11 expression and reduces lipid ROS accumulation, thereby promoting tumor growth and ferroptosis resistance [81].

The period circadian regulator PER1 also contributes significantly to ferroptosis [24]. Overexpression of PER1 reduces HIF-1α levels, promotes ROS accumulation, and accelerates lipid peroxidation, thereby increasing ferroptosis. Silencing PER1 leads to ferroptosis resistance and accelerated tumor progression in xenograft models. Other oncogenic regulators modulate ferroptosis in OSCC. Adipocyte enhancer-binding protein 1 (AEBP1) activates the JNK/p38/ERK pathway, suppressing lipid peroxidation and protecting cisplatin-treated OSCC cells from ferroptosis; knockdown of AEBP1 restores ferroptotic sensitivity [83]. PPT1 upregulates GPX4 and inhibits ferroptosis, correlating with poor patient outcomes [32]. Cytokeratin 19 (CK19) also functions as a ferroptosis inhibitor by suppressing ACSL4 activity, and its silencing induces ferroptosis, reducing OSCC migration and viability [54]. Receptor accessory protein 6 (REEP6) was recently identified as a ferroptosis suppressor in OSCC, where its overexpression maintains endoplasmic reticulum homeostasis by regulating ACSL4 and confers resistance to ferroptosis inducers [90]. CDH4 (R-cadherin) was also found to be upregulated in OSCC, where its overexpression promoted proliferation, migration, and EMT while reducing ferroptosis sensitivity through enhancing GPX4 and GSH levels and lowering lipid peroxidation, thereby correlating with poor patient survival [84]. These findings illustrate that OSCC progression involves active repression of ferroptosis by both oncogenic and lineage-specific regulators.

### 3.3. Non-Coding RNAs and Epigenetic Modulation

Non-coding RNAs (ncRNAs) represent an additional layer of ferroptosis regulation in OSCC. MicroRNAs such as miR-34c-3p promote ferroptosis by suppressing GPX4 and increasing ROS, whereas miR-520d-5p is sponged by circular RNA circFNDC3B, leading to increased SLC7A11 expression and ferroptosis inhibition [96,97]. Similarly, miR-26a-5p was shown to enhance OSCC sensitivity to erastin by directly targeting the 3′UTR of SLC7A11, thereby reducing cystine uptake, depleting GSH, and promoting ferroptosis [60]. Long non-coding RNAs (lncRNAs) also contribute; prognostic signatures incorporating ferroptosis-related lncRNAs (FRLs) such as STARD4-AS1, MIAT, AC099850.3, and AC090246.1 stratify OSCC patients into high- and low-risk groups, correlating with survival and immune infiltration patterns [29]. Silencing of STARD4-AS1 enhances ferroptosis and suppresses proliferation, highlighting its functional relevance [29].

Circular RNAs play a particularly strong role in drug resistance. circ_0000140, upregulated in cisplatin-resistant OSCC, suppresses ferroptosis by sponging miR-527 and releasing SLC7A11 from inhibition [30]. Knockdown of circ_0000140 promotes ferroptosis and restores cisplatin sensitivity, while miR-527 silencing or SLC7A11 re-expression rescues the resistant phenotype. Epigenetic modifications also regulate ferroptosis in OSCC; RNA methyltransferase METTL3 stabilizes SLC7A11 mRNA through *N*6-methyladenosine (m^6^A) modification, enhancing ferroptosis resistance [85,86,98]. In contrast to METTL3, the m^6^A demethylase FTO was shown to sensitize OSCC cells to ferroptosis by destabilizing ACSL3 and GPX4 transcripts, thereby reducing anti-ferroptotic defenses and enhancing ferroptotic vulnerability in vitro and in vivo [87]. Together, these findings highlight the importance of ncRNAs and epigenetic regulators in modulating ferroptotic vulnerability in oral cancer.

### 3.4. Metabolic Enzymes and Ferroptosis Sensitivity

Metabolic enzymes strongly influence ferroptosis in OSCC. TPI1, a glycolytic enzyme, is upregulated in cisplatin-resistant OSCC and suppresses ferroptosis by reducing ROS and lipid peroxidation [31]. Silencing TPI1 increases free iron and lipid ROS, promotes ferroptosis, and restores cisplatin sensitivity both in vitro and in xenograft models. This positions TPI1 as both a prognostic biomarker and a therapeutic target.

Lipid metabolism enzymes also contribute. ACSL4 and LPCAT3 facilitate the incorporation of PUFAs into membrane phospholipids, increasing susceptibility to ferroptosis [49]. Conversely, reduced ACSL4 expression correlates with poor prognosis and diminished ferroptotic sensitivity in OSCC cohorts [54]. Phospholipase A2 group VI (iPLA2β) counters ferroptosis by cleaving oxidized PUFA chains from membranes, thereby reducing lipid peroxidation and cell death [48,99]. Mitochondrial metabolism also regulates ferroptosis. Inhibition of dynamin-related protein 1 (DRP1), a mediator of mitochondrial fission, increases ferroptosis sensitivity in OSCC, indicating that mitochondrial dynamics influence lipid peroxidation and cell fate [100]. These findings demonstrate that ferroptosis sensitivity is tightly integrated with metabolic plasticity in oral cancer.

### 3.5. Chemoresistance and Ferroptosis in OSCC

Cisplatin resistance represents one of the most significant clinical challenges in OSCC. Resistant cells exhibit lower baseline ROS and lipid peroxidation compared to sensitive cells, along with upregulation of antioxidant pathways such as Nrf2 and GPX4 [26]. Pharmacological ferroptosis inducers restore cisplatin sensitivity: erastin blocks system Xc^−^, RSL3 inhibits GPX4, and both enhance ferroptotic death in resistant OSCC cells [26,31,83,101,102,103]. Non-coding RNAs are also implicated in chemoresistance. circ_0000140 promotes cisplatin resistance by repressing ferroptosis, and its inhibition restores drug sensitivity [30]. Natural products such as *Evodia lepta* extract downregulate GPX4 and HSPA5, selectively inducing ferroptosis in cisplatin-resistant OSCC cells [34]. These findings highlight ferroptosis induction as a promising strategy to overcome chemotherapy resistance in oral cancer.

### 3.6. Tumor Microenvironment and Ferroptosis

TME significantly affects ferroptosis in OSCC. High FPscore correlates with increased infiltration of CD8^+^ T cells, dendritic cells, and NK cells, as well as with better responses to immune checkpoint blockade [94]. PDPN^+^ cancer-associated fibroblasts were shown to transfer exosomal lncRNA FTX to OSCC cells, activating an FTX/FEN1/ACSL4 axis that suppresses ferroptosis and enhances cell motility, linking CAF signaling with ferroptosis resistance and poor prognosis [89]. Ferroptotic cancer cells can actively modulate the immune landscape by releasing damage-associated molecular patterns (DAMPs) such as HMGB1, ATP, and oxidized phospholipids, which act as immunogenic signals that recruit and activate dendritic cells and CD8^+^ cytotoxic T cells [104]. This process enhances antitumor immunity and improves the efficacy of immune checkpoint inhibitors. However, ferroptosis may also promote immunosuppression under certain contexts. Lipid peroxidation-derived aldehydes and oxidized phosphatidylethanolamines can impair dendritic cell antigen presentation or induce myeloid-derived suppressor cells (MDSCs), thereby dampening immune responses [105,106]. Recent data indicate that tumor-associated macrophages exposed to ferroptotic debris may adopt an M2-like phenotype, supporting tumor progression. These findings suggest that ferroptosis-driven immune signaling is highly context-dependent, and therapeutic ferroptosis induction should be carefully integrated with immunotherapy to maximize synergistic efficacy while minimizing immune suppression.

### 3.7. Dual Roles of Ferroptosis in Tumorigenesis

Ferroptosis exhibits a Janus-faced role in cancer biology, acting as both a tumor suppressor and a potential promoter of tumorigenesis depending on the microenvironmental and genetic context. On one hand, ferroptotic cell death limits cancer cell survival, eliminates therapy-resistant clones, and promotes immunogenic clearance. On the other hand, persistent lipid peroxidation and chronic sublethal ferroptotic stress can generate pro-inflammatory signals that promote mutagenesis, angiogenesis, and metastatic adaptation [20]. In OSCC, chronic oxidative stress in tobacco- and alcohol-related lesions may trigger partial ferroptotic signaling that enhances tumor evolution. Therefore, the therapeutic induction of ferroptosis should be optimized to achieve complete cytotoxicity rather than incomplete sublethal lipid peroxidation. This duality underscores the need for precise control of ferroptosis intensity and timing in clinical applications.

### 3.8. Natural Products and Nanomedicine as Ferroptosis Inducers

Natural compounds provide a rich source of ferroptosis inducers in OSCC. Piperlongumine reduces GPX4 and SLC7A11 expression, enhances ROS and lipid peroxidation, and suppresses proliferation in OSCC cells [33]. Its effects are reversed by the ferroptosis inhibitor ferrostatin-1 and the antioxidant N-acetylcysteine (NAC), confirming ferroptosis dependence. PL activity is synergistically enhanced when combined with the glutaminase inhibitor CB-839, which depletes glutathione and further increases lipid peroxidation. Quercetin and trifluoperazine also promote ferroptosis in OSCC models, reducing tumor cell viability [107,108].

Nanotechnology approaches expand the therapeutic landscape by enhancing targeted delivery and reducing systemic toxicity. For example, photothermal-responsive manganese dioxide nanoprobes generate ROS, deplete glutathione, and release encapsulated RSL3 under near-infrared irradiation, triggering robust ferroptosis in OSCC cells [109]. Fluorescent hydrogel films incorporating carbon dots detect Fe^3+^ ions and semi-quantify ferroptosis in OSCC, serving as both diagnostic and therapeutic platforms [110]. These strategies highlight the potential of integrating ferroptosis modulation into precision medicine approaches for oral cancer.

In summary, preclinical evidence establishes ferroptosis as a suppressed but targetable pathway in OSCC. Tumor suppressors such as p53 and PER1, oncogenes such as AEBP1 and PPT1, and non-coding RNAs including circ_0000140 and STARD4-AS1 critically shape ferroptosis susceptibility. Metabolic enzymes and mitochondrial dynamics further modulate this balance, while ferroptosis induction offers a strategy to overcome cisplatin resistance. Interactions with the tumor microenvironment suggest both opportunities and challenges for immunotherapy integration. Natural compounds and nanomedicine platforms provide innovative tools to exploit ferroptosis in OSCC, paving the way for future clinical translation.

## 4. Ferroptosis and Therapy Resistance in Oral Cancer

Therapeutic resistance remains a major obstacle in the clinical management of OSCC. Increasing evidence suggests that ferroptosis is closely intertwined with mechanisms of resistance to chemotherapy, radiotherapy, and immunotherapy, thereby providing a mechanistic framework for overcoming treatment failure.

### 4.1. Chemotherapy Resistance

Cisplatin is the backbone of OSCC chemotherapy, but resistant cells exhibit lower basal lipid peroxidation and ROS, accompanied by upregulation of GPX4 and SLC7A11, which collectively suppress ferroptosis [26]. Induction of ferroptosis by erastin (system Xc^−^ inhibitor) or RSL3 (GPX4 inhibitor) restores lipid ROS and sensitizes resistant OSCC cells to cisplatin both in vitro and in vivo models [61]. Non-coding RNAs further contribute: circ_0000140 promotes cisplatin resistance by sponging miR-527, thereby upregulating SLC7A11, whereas its silencing reactivates ferroptosis and restores cisplatin sensitivity [30]. Metabolic reprogramming also plays a role, as TPI1 is elevated in resistant OSCC and suppresses ferroptosis; knockdown of TPI1 increases lipid ROS and reverses resistance [31]. Natural products such as *Evodia lepta* extract induce ferroptosis via GPX4 and HSPA5 suppression, selectively killing cisplatin-resistant OSCC cells and reducing PD-L1 expression [34]. In addition, TCF12 was recently identified as a cisplatin-sensitizing factor in OSCC by repressing OTUB1-mediated deubiquitination of SLC7A11, thereby promoting ferroptosis both in vitro and in vivo models [61]. Drug-tolerant persister OSCC cells generated under cisplatin exposure exhibit elevated FSP1 and lipid metabolism signatures, and inhibition of FSP1 with iFSP1 reactivated ferroptosis, reduced stemness, and overcame chemoresistance in patient-derived xenograft models [79]. Collectively, these findings indicate that ferroptosis suppression is a hallmark of cisplatin resistance, and its reactivation is a promising strategy for chemosensitization. In addition to transcriptional and metabolic regulation, ubiquitination has recently emerged as a key post-translational mechanism modulating ferroptosis-related proteins such as *ACSL4* and p62, thereby influencing lipid peroxidation and drug resistance in OSCC [111]. This layer of regulation provides novel druggable nodes within the ferroptosis network, linking protein stability and degradation to redox homeostasis and cell death control.

### 4.2. Radiotherapy Resistance

Radiotherapy exerts part of its cytotoxicity through ROS generation, which overlaps with lipid peroxidation processes characteristic of ferroptosis [112]. Experimental evidence in head and neck cancer cells shows that radiation increases lipid ROS and ferroptosis markers, and ferroptosis inhibition with ferrostatin-1 attenuates this effect [113,114]. Conversely, ferroptosis inducers enhance radiation-mediated tumor cell death, suggesting that ferroptosis contributes to radiosensitization [113]. Regulators such as Nrf2 and GPX4 confer radioresistance by reinforcing antioxidant defenses, whereas ACSL4 and LPCAT3 promote radiosensitivity by enriching PUFA-PL for peroxidation [87,115]. Nanoparticle-based approaches have been designed to combine radiotherapy with ferroptosis induction, amplifying ROS and overcoming radioresistance in OSCC models [116]. In line with these findings, manganese was recently shown to promote ferroptosis in OSCC by inducing YAP/TAZ phase separation and activating ACSL4, with higher expression of this axis correlating with improved patient prognosis [53]. In support of this concept, astaxanthin was recently shown to synergize with ionizing radiation in OSCC by inhibiting GPX4 and SLC7A11, enhancing ACSL4 expression, and thereby amplifying ferroptosis and radiosensitivity in both cell and xenograft models [117].

### 4.3. Immunotherapy Resistance

Immune checkpoint blockade has limited efficacy in OSCC, with response rates of 15–20% [118]. Recent transcriptomic studies show that higher ferroptosis activity correlates with increased infiltration of CD8^+^ T cells and higher expression of checkpoint molecules, implying that ferroptosis may shape immunotherapy responsiveness [94]. Induction of ferroptosis in OSCC not only triggers tumor cell death, but also decreases PD-L1 expression [34], potentially improving the efficacy of anti–PD-1/PD-L1 therapy. However, ferroptosis can also have paradoxical effects, as lipid peroxidation products may impair dendritic cell function or promote recruitment of myeloid-derived suppressor cells, and ferroptosis of CD8^+^ T cells in the tumor microenvironment may reduce cytotoxic immunity [76,93]. More recently, a CRISPR-based synthetic lethality screen identified an intrinsic role of PD-L1 in conferring ferroptosis resistance in HNSCC, mediated through transcriptional activation of SOD2; PD-L1-deficient cells displayed heightened susceptibility to ferroptosis and immunogenic cell death, suggesting that intrinsic PD-L1 functions shape ferroptosis vulnerability beyond immune checkpoint signaling [88]. Thus, therapeutic strategies must balance induction of tumor ferroptosis with preservation of immune effector functions.

### 4.4. Genomic and Transcriptomic Insights into Ferroptosis-Related Prognosis in OSCC

Recent multi-omics studies have identified ferroptosis-related gene (FRG) signatures as prognostic indicators in oral squamous cell carcinoma. Weighted gene co-expression network analysis (WGCNA) of TCGA-OSCC datasets has identified key FRGs such as *SLC7A11*, *GPX4*, *ACSL4*, *TFRC*, and *FTH1* as determinants of patient survival and immune infiltration [76]. A seven-gene prognostic model constructed using Cox and LASSO regression effectively stratified patients into high- and low-risk groups, with lower ferroptosis scores correlating with immunosuppressive TMEs and poor survival outcomes. Similarly, a ten-lncRNA ferroptosis-related signature, including STARD4-AS1, was proposed as an independent predictor of prognosis, where high-risk cohorts exhibited reduced ferroptosis activity and greater resistance to immunotherapy [29]. These integrative analyses underscore the clinical relevance of ferroptosis regulators as potential biomarkers and therapeutic targets in OSCC [93].

In summary, unlike the basic preclinical findings summarized earlier, the clinical relevance of ferroptosis lies in its ability to reprogram therapy-resistant phenotypes. By targeting ferroptosis, it is possible to restore chemosensitivity, enhance radiosensitivity, and modulate immune responses. This highlights ferroptosis as a promising integrative approach to overcome resistance and improve therapeutic outcomes in OSCC.

## 5. Therapeutic Strategies Targeting Ferroptosis in Oral Cancer

**Figure 3:**
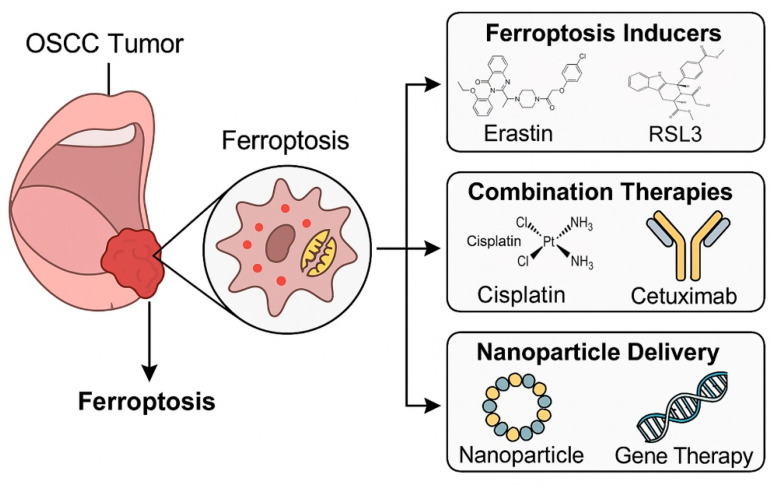
Therapeutic opportunities targeting ferroptosis in OSCC. Small-molecule inducers such as erastin and sulfasalazine inhibit system Xc^−^, while RSL3 and FIN56 directly inactivate GPX4. Repurposed drugs, including sorafenib, artesunate, and trifluoperazine, induce ferroptosis by disrupting antioxidant defenses. Natural products such as piperlongumine, *Evodia lepta* extract, and quercetin promote lipid peroxidation and suppress GPX4 or SLC7A11. Nanomedicine-based delivery platforms amplify ROS, deplete GSH, and enable tumor-selective ferroptosis induction. Combination strategies with cisplatin, radiotherapy, or immune checkpoint inhibitors yield synergistic effects, underscoring ferroptosis as a clinically relevant therapeutic avenue.

Mounting preclinical evidence has established ferroptosis as a central vulnerability in OSCC. The challenge now is translating these insights into therapeutic strategies that can be clinically deployed. Targeting ferroptosis involves either promoting pro-ferroptotic pathways, such as iron accumulation, lipid peroxidation, and inhibition of antioxidant defenses, or suppressing anti-ferroptotic mechanisms including GPX4, SLC7A11, FSP1, and Nrf2. In OSCC, several categories of interventions have emerged: small-molecule ferroptosis inducers, repurposed drugs, natural compounds, nanomedicine-based delivery systems, and rational combination strategies (Figure 3 and Table 2). This section provides a comprehensive overview of therapeutic approaches aimed at leveraging ferroptosis in oral cancer.

### 5.1. Small-Molecule Ferroptosis Inducers

The cystine/glutamate antiporter system Xc^−^, composed of SLC7A11 and SLC3A2, is the primary supplier of cysteine for GSH synthesis. Inhibitors of system Xc^−^, such as erastin and sulfasalazine, induce ferroptosis by depleting GSH and inactivating GPX4 [134]. In OSCC cell lines, pharmacological blockade of system Xc^−^ leads to lipid ROS accumulation, reduced proliferation, and increased apoptosis–ferroptosis hybrid death [97]. Sulfasalazine, a clinically approved anti-inflammatory drug, has shown selective cytotoxicity against OSCC cells with minimal effects on normal fibroblasts [119]. These findings suggest that system Xc^−^ inhibitors may be repurposed for OSCC therapy, particularly in patients with high SLC7A11 expression.

GPX4 is the central enzymatic suppressor of ferroptosis, reducing PL hydroperoxides to their corresponding alcohols [12]. Small molecules such as RSL3, ML210, and FIN56 irreversibly inactivate GPX4, triggering lipid peroxidation and ferroptotic death [135]. OSCC cells are highly dependent on GPX4 for survival, especially under conditions of metabolic stress or EMT [136]. In CAL27 and SCC9 cells, RSL3 treatment results in mitochondrial shrinkage, ROS accumulation, and ferroptosis-specific cell death that is rescued by ferrostatin-1, confirming GPX4 as a therapeutic target [87]. While clinical-grade GPX4 inhibitors remain under development, the preclinical data support their potential as selective OSCC therapeutics.

Iron chelators such as deferoxamine (DFO) protect against ferroptosis by reducing labile Fe^2+^, whereas iron donors or modulators of ferritinophagy enhance ferroptotic sensitivity. In OSCC, increased transferrin receptor 1 (TfR1) expression and reduced ferroportin contribute to an expanded labile iron pool, sensitizing cells to ferroptosis induction [17,45]. Agents that promote ferritinophagy via NCOA4 activation or lysosomal autophagy increase intracellular iron release, triggering lipid ROS accumulation. Preclinical studies combining iron donors with GPX4 inhibitors show synergistic ferroptotic death in OSCC cells, though clinical translation requires careful balancing to avoid systemic iron toxicity [54,108].

### 5.2. Repurposed Drugs with Ferroptosis-Inducing Activity

Several drugs already in clinical use for non-cancer indications have been found to induce ferroptosis and are being investigated in OSCC models. By inhibiting HMG-CoA reductase, statins reduce the synthesis of mevalonate-derived coenzyme Q_10_ (CoQ_10_), a lipid antioxidant. This limits the activity of both GPX4 and FSP1, sensitizing cancer cells to ferroptosis in several tumor types [17,20]. While direct evidence in OSCC is still lacking, the high dependence of OSCC cells on SLC7A11 and GPX4 provides a strong rationale for further exploration of statin repurposing. Artesunate generates ROS via iron-dependent reactions, effectively inducing ferroptosis in OSCC cell lines [66]. Studies demonstrate that artesunate treatment increases intracellular iron levels, lipid ROS, and synergizes with cisplatin to suppress tumor growth [32]. Sorafenib originally developed as a multikinase inhibitor, also functions as a system Xc^−^ inhibitor [65]. In OSCC, sorafenib enhances cisplatin sensitivity and induces ferroptosis, effects that are abrogated by ferrostatin-1 [120]. More recently, TFP, an FDA-approved antipsychotic, has been repurposed as a ferroptosis inducer in oral cancer [108]. TFP acts via autophagy activation, inhibition of the SLC7A11/GPX4 axis, and mitochondrial damage. Molecular docking studies confirmed direct GPX4 inhibition, and high GPX4 expression in oral cancer biopsies correlated with poor prognosis, highlighting TFP as a promising repositioned drug candidate.

Other agents are emerging as additional repositioning opportunities. Disulfiram, an alcohol aversion drug, particularly in its copper-complexed form, activates ferroptosis through modulation of the Nrf2/HO-1 pathway in oral cancer cells [67]. Quisinostat, a HDAC inhibitor, sensitizes OSCC to ferroptosis by disrupting redox homeostasis and enhancing lipid peroxidation [121]. Carnosic acid, a dietary diterpene, induces ferroptosis in cisplatin-resistant OSCC cells, suggesting its potential as a repositioned chemosensitizer [102]. Finally, melatonin, widely used as a sleep-regulating agent, potentiates erastin-induced ferroptosis in OSCC by amplifying ROS accumulation and mitochondrial stress [122]. Lanperisone, a muscle relaxant, and trigonelline, a natural alkaloid initially investigated in metabolic disorders, have been suggested in recent reviews as potential ferroptosis-inducing candidates in OSCC, although direct experimental evidence remains limited [14,137].

Together, these findings demonstrate that a broad spectrum of repurposed agents—including cardiovascular drugs, antimalarials, kinase inhibitors, antipsychotics, HDAC inhibitors, and metabolic modulators—converge on ferroptosis pathways in OSCC. Leveraging their established pharmacological profiles may accelerate the clinical translation of ferroptosis-based strategies in oral cancer.

### 5.3. Natural Products Targeting Ferroptosis

Natural compounds provide a diverse source of ferroptosis inducers with multi-target mechanisms in OSCC. Piperlongumine, a plant alkaloid, decreases GPX4, FTH1, and SLC7A11 expression while upregulating DMT1, thereby promoting iron accumulation and lipid peroxidation in OSCC cells [33]. Its effects are reversed by ferrostatin-1 and NAC, confirming ferroptosis dependence. Importantly, PL acts synergistically with the glutaminase inhibitor CB-839 to enhance glutathione depletion and increase lipid ROS beyond monotherapy, providing proof-of-concept for natural product-based combination regimens. *Evodia lepta* extract selectively induces ferroptosis in tongue squamous cell carcinoma by downregulating GPX4 and HSPA5, while simultaneously reducing PD-L1 expression [34]. This dual effect suggests both cytotoxic and immunomodulatory benefits, particularly in cisplatin-resistant OSCC where cisplatin itself paradoxically upregulates GPX4. Quercetin has also been shown to potentiate ferroptosis by suppressing SLC7A11, reducing intracellular glutathione, and increasing lipid peroxidation [107]. Its action is partly mediated by inhibition of the mTOR/S6KP70 signaling axis, and co-treatment with mTOR inhibitors further augments ferroptosis in OSCC cells. In addition, TFP, while classically categorized as a repurposed antipsychotic, has been reported in the context of natural compounds to inhibit GPX4, elevate lipid ROS, and promote autophagy-dependent ferroptosis [108]. Brusatol, a quassinoid from *Brucea javanica*, induces ferroptosis in OSCC by inhibiting the Nrf2/GCLC pathway, leading to SLC7A11 suppression, GSH depletion, Fe^2+^/ROS accumulation, and Cal-27 tumor growth inhibition [123]. Resveratrol was also shown to accelerate ferroptosis in OSCC by promoting p53 nuclear translocation, suppressing SLC7A11 expression, depleting GSH, and increasing Fe^2+^ and ROS, thereby inhibiting tumor growth and malignant behaviors [82].

Fucoxanthin, a marine carotenoid, was also reported to induce ferroptosis in SCC-25 tongue squamous carcinoma cells by downregulating the Nrf2/HO-1/GPX4 pathway and increasing ROS, iron accumulation, and p53 expression, thereby suppressing tumor cell viability [124]. Baicalin was also reported to promote ferroptosis in OSCC by directly suppressing FTH1 expression, thereby inhibiting proliferation, invasion, and epithelial–mesenchymal transition, highlighting FTH1 as a potential therapeutic target [125]. Aqueous-soluble components of sporoderm-removed *Ganoderma lucidum* spore powder (A-GSP) were also reported to promote ferroptosis in OSCC by inducing Fe^2+^ influx, depleting GSH, upregulating ACSL4, and downregulating GPX4, resulting in mitochondrial dysfunction and tumor suppression in xenograft models [126]. Its inclusion highlights the overlap between repurposed drugs and natural derivatives in ferroptosis modulation. Collectively, these findings underscore the therapeutic promise of natural alkaloids and plant-derived compounds as ferroptosis inducers in OSCC, either alone or in rational combination strategies.

### 5.4. Nanomedicine-Based Ferroptosis Strategies

Nanotechnology offers innovative strategies to deliver ferroptosis inducers, amplify oxidative stress, and overcome drug resistance in OSCC. Hollow mesoporous manganese dioxide nanoparticles loaded with RSL3 and conjugated with tumor-targeting peptides generate ROS and deplete GSH upon near-infrared irradiation. In OSCC models, this photothermal approach induces “explosive” ferroptosis and suppresses tumor growth in vivo [109]. Carbon dot–based hydrogels have been developed as theranostic systems that can detect Fe^3+^ at nanomolar concentrations and simultaneously promote ferroptosis in OSCC cells [110]. Similarly, iron-doped dopamine composites catalyze Fenton-like reactions, boosting hydroxyl radical generation and lipid peroxidation in OSCC models [127].

Additional nanoplatforms are emerging with unique mechanisms. Zero-valent iron nanoparticles (ZVI NPs) trigger mitochondrial lipid peroxidation and reduce GPX activity, thereby initiating ferroptosis in OSCC cells, although resistant subclones adapt via enhanced antioxidant capacity [138]. Sorafenib–chlorin e6 nanoparticles (Sor-Ce6 NPs), created via carrier-free self-assembly with Fe^3+^, combine ferroptosis induction with photodynamic therapy, significantly enhancing antitumor activity while overcoming hypoxia-related resistance [120]. Another strategy involves DMEFe nanoparticles, where doxorubicin-loaded mesoporous silica particles are coated with a metal polyphenol layer; these systems exhibit pH-sensitive release, increase ROS levels, and modulate ferroptosis-related genes, resulting in potent antitumor efficacy in OSCC models [139].

More recently, metal ion interference nanoplatforms such as Zn@CDDP@HMON have been engineered to strategically release Zn^2+^ and Pt^2+^ in the tumor microenvironment [140]. This dual-ion release enhances ROS production through NADPH oxidases and Fenton-like reactions while depleting GSH and inhibiting GPX4, sensitizing OSCC cells to ferroptosis. These nanoplatforms also enable T1-MRI imaging for real-time monitoring. Furthermore, exosome-encapsulated ultrasmall AuMn nanoclusters (Exo-AMNCs) combine the intrinsic ROS-generating properties of AuMn alloys with the targeting and biocompatibility of NK cell–derived exosomes, achieving selective ferroptosis induction and simultaneous fluorescence imaging in OSCC models [128]. In addition, CD44-targeted mP6/Rg3 micelles were recently shown to inhibit ABCB1, promote ferroptosis in oral cancer stem cells, and suppress OSCC proliferation and migration in vitro and in vivo [129].

Collectively, these diverse nanomedicine strategies improve tumor selectivity, minimize systemic toxicity, and provide multifunctional platforms that integrate diagnosis and therapy, underscoring their translational potential for ferroptosis-based clinical interventions in OSCC.

### 5.5. Combination Strategies Involving Ferroptosis

Combination approaches that integrate ferroptosis modulation with existing therapies are gaining increasing attention in OSCC. Notably, in silico network pharmacology analyses have further highlighted GSH as a central regulator of ferroptosis in oral cancer, identifying 14 potential molecular targets including EGFR, PTGS2, HIF1A, and SLC3A2, and supporting the rationale for GSH modulation as part of ferroptosis-based therapeutic strategies [141].

**Chemotherapy combinations.** Combining ferroptosis inducers with cisplatin effectively overcomes resistance in OSCC. Erastin or RSL3 restores cisplatin sensitivity in resistant cells, while natural compounds such as *Evodia lepta* extract synergize with cisplatin to reduce tumor growth [34]. Mechanistic studies have shown that circ_0000140 suppresses ferroptosis through the miR-527/SLC7A11 axis, thereby promoting cisplatin resistance, whereas circ_0000140 knockdown restores ferroptosis and sensitizes cells to cisplatin [30]. Similarly, carnosic acid reverses cisplatin resistance by inactivating the Nrf2/HO-1 pathway and inducing ferroptosis [102]. Beyond these, amoxicillin, a widely used antibiotic, has been reported to enhance cisplatin efficacy in OSCC by inducing mitochondrial dysfunction and ferroptosis [130]. Collectively, these findings underscore ferroptosis as a central determinant of cisplatin responsiveness.

**Radiotherapy combinations.** Ionizing radiation produces ROS and lipid radicals, initiating ferroptosis. Ferroptosis inhibitors such as ferrostatin-1 suppress radiation-induced cell death, whereas inducers enhance radiosensitivity [113]. Nanoparticle-based platforms amplify lipid peroxidation to synergize with radiotherapy in OSCC models [109]. Additionally, manganese has been shown to drive ferroptosis via YAP/TAZ-mediated activation of ACSL4, thereby potentiating the effects of radiation [53]. In line with these findings, hyperbaric oxygen was recently demonstrated to synergize with X-ray irradiation in OSCC by suppressing GPX4 expression, enhancing ferroptosis, and re-sensitizing radio-resistant tumor cells in both xenograft models and patient samples [131].

**Photodynamic therapy combinations.** Beyond conventional chemo- and radiotherapy, ferroptosis has also been leveraged to enhance photodynamic therapy (PDT), where a Ce6–erastin supramolecular nanodrug synergistically relieved hypoxia, inhibited SLC7A11, and produced sustained ROS through Fenton chemistry, yielding potent antitumor effects in tongue OSCC models [132].

**Immunotherapy combinations.** Ferroptosis enhances tumor immunogenicity by releasing damage-associated molecular patterns and oxidized lipids, which stimulate dendritic cell maturation and T-cell activation [104]. In OSCC, ferroptosis induction reduces PD-L1 expression, potentially improving responses to immune checkpoint inhibitors [34]. Nevertheless, ferroptosis can also generate immunosuppressive lipid mediators or induce ferroptosis in CD8^+^ T cells within the tumor microenvironment, complicating outcomes [76,93]. Adding to these findings, a ferroptosis-associated gene signature (FGS) developed in HNSCC, validated also in OSCC samples, was shown to predict overall survival and immunotherapy responsiveness, with links to CD276-driven immunosuppressive fibroblasts and ATG5-mediated T cell exclusion, highlighting the clinical utility of ferroptosis-based prognostic models [142]. Integration of ferroptosis with immunotherapy thus requires careful balancing, and biomarker-driven stratification may help identify patients most likely to benefit [94,133].

**Multi-modal combinations.** Preclinical evidence suggests that ferroptosis interacts with other RCD pathways such as apoptosis, autophagy, and pyroptosis. RSL3, a GPX4 inhibitor, was found to also activate autophagy, and its combination with the autophagy inducer LYN-1604 synergistically inhibited OSCC cell proliferation and migration [36]. Such dual-induction strategies may be especially effective against persister and mesenchymal-like OSCC cells that evade apoptosis and resist conventional therapies.

In summary, therapeutic strategies targeting ferroptosis in OSCC span multiple approaches, including direct inhibition of system Xc^−^ and GPX4, repurposing of agents such as sorafenib and trifluoperazine, and natural products like piperlongumine and *Evodia lepta*. Nanomedicine-based platforms provide targeted delivery, diagnostic integration, and improved safety. Combination regimens with chemotherapy, radiotherapy, and immunotherapy highlight ferroptosis as a critical axis to overcome therapy resistance. Moreover, multimodal strategies involving autophagy or pyroptosis expand the therapeutic window. Together, these approaches underscore the translational potential of ferroptosis modulation in oral cancer and provide a strong rationale for biomarker-guided clinical trials.

## 6. Ferroptosis in Oral Diseases Beyond Cancer

While the majority of ferroptosis studies involving the oral cavity have focused on OSCC, recent investigations suggest that ferroptosis also contributes to non-malignant oral diseases, including pulpitis, periodontitis, and infection-associated inflammation. Beyond malignancy, ferroptosis contributes to inflammatory and infectious oral diseases [105], supporting its relevance across the spectrum of oral pathology. These findings expand the scope of ferroptosis from cancer biology to broader oral pathophysiology, highlighting both its pathogenic and therapeutic implications.

### 6.1. Pulpal and Endodontic Diseases

Dental pulp inflammation (pulpitis) is characterized by severe nociceptive signaling, vascular changes, and progressive tissue degeneration. Classical inflammatory cytokines such as IL-1β and TNF-α are upregulated in inflamed pulp tissue, and recent studies have begun to link these processes with ferroptotic cell death [143]. A landmark investigation demonstrated that EZH2 knockout in pulpitis-affected dental pulp vascular endothelial cells altered STAT3 methylation and regulated GPX4 expression, thereby modulating ferroptosis and exacerbating oxidative stress [144]. These findings provide direct evidence that ferroptosis contributes to the pathogenesis of pulpitis. Earlier studies in non-oral tissues also suggested that p53–SLC7A11/GPX4 signaling may connect inflammation and ferroptosis [145]. Together, these findings indicate that ferroptosis contributes to pulpitis pathogenesis and may represent a novel therapeutic target [105].

Epigenetic regulation further modulates pulpitis. Competing endogenous RNA (ceRNA) networks involving ferroptosis-related lncRNAs and miRNAs have been constructed in irreversible pulpitis, linking ncRNA regulation to ferroptotic signaling [146]. These studies highlight the relevance of ferroptosis in the context of dental pulp biology, though more direct mechanistic validation is needed.

### 6.2. Periodontitis

Periodontitis is a chronic inflammatory disease marked by alveolar bone resorption and the destruction of periodontal supporting tissues. Evidence indicates that ferroptosis contributes to oxidative stress-induced periodontal cell injury. In gingival fibroblasts and periodontal ligament cells, elevated iron and lipid ROS promote ferroptotic cell death, which exacerbates inflammatory cascades [147]. In vivo models of periodontitis demonstrate increased expression of ferroptosis-associated genes such as *ACSL4*, *PTGS2*, and *TFRC*, linking ferroptosis with periodontal tissue destruction [148].

Beyond preclinical findings, a recent multi-platform clinical study of gingival crevicular fluid and gingival single-cell transcriptomes in periodontitis patients revealed distinct ferroptosis-associated protein signatures, including *SNCA*, *FTH1*, *HSPB1*, *CD44*, and *GCLC*, and highlighted fibroblasts as particularly susceptible to ferroptosis, thereby directly implicating this cell death pathway in disease progression [149]. Furthermore, immune cell interactions within periodontal lesions also appear to be modulated by ferroptosis; macrophages undergoing ferroptosis exhibit impaired immune clearance and dysregulated iron recycling, fueling chronic inflammation [150,151]. Collectively, these insights suggest that ferroptosis is not only a driver of periodontal tissue damage but may also represent a novel therapeutic target to mitigate disease progression.

### 6.3. Infection, Inflammation, and Immunity in the Oral Cavity

Beyond pulpitis and periodontitis, ferroptosis has been implicated in infection- and inflammation-related oral conditions. Neutrophils exposed to bacterial pathogens undergo ferroptosis-like lipid peroxidation, releasing neutrophil extracellular traps (NETs) that both limit infection and contribute to collateral tissue damage [152,153]. Moreover, macrophages in oral lesions phagocytose senescent erythrocytes, releasing iron and fueling ferroptotic cascades that modulate immune homeostasis [150].

These processes suggest that ferroptosis operates at the intersection of infection, inflammation, and immunity in the oral cavity. While it may contribute to host defense by eliminating damaged cells, excessive ferroptosis may exacerbate inflammatory injury, underscoring the importance of balance in therapeutic targeting.

### 6.4. Implications for Oral Disease Therapy

The involvement of ferroptosis in non-malignant oral diseases opens new therapeutic avenues. Antioxidants such as ferrostatin-1 or liproxstatin-1 could potentially mitigate pulpitis- or periodontitis-associated tissue destruction by inhibiting ferroptotic cascades [148]. Conversely, controlled ferroptosis induction might enhance clearance of infected or dysregulated cells in chronic inflammatory lesions [105,143]. Translating these concepts requires careful context-specific evaluation, as ferroptosis may have dual roles in promoting both host defense and tissue injury.

In summary, ferroptosis extends beyond oral cancer into diverse non-malignant oral diseases, including pulpitis, periodontitis, and infection-associated inflammation. In these contexts, ferroptosis contributes to oxidative stress, cytokine release, and immune dysregulation, influencing disease progression and tissue outcomes. While still underexplored, these findings suggest that ferroptosis modulation—either inhibition to protect tissues or induction to eliminate damaged cells—may represent a novel therapeutic strategy in oral disease management [105].

## 7. Conclusions and Perspectives

Ferroptosis has rapidly evolved from a mechanistic curiosity to a compelling therapeutic concept in oral cancer research. In OSCC, ferroptosis integrates iron metabolism, lipid peroxidation, and antioxidant defenses, and its suppression underlies many of the hallmarks of tumor progression, including metabolic plasticity, EMT, and resistance to chemotherapy. Preclinical studies demonstrate that targeting ferroptosis not only restores sensitivity to cisplatin but also enhances the efficacy of radiotherapy and immunotherapy, positioning this pathway as a unifying vulnerability across treatment modalities. Beyond oncology, ferroptosis contributes to non-malignant oral diseases such as pulpitis and periodontitis, where its excessive activation promotes oxidative tissue damage (Table 3).

Clinically, these insights open several opportunities. Ferroptosis inducers, ranging from small-molecule GPX4 inhibitors and system Xc^−^ blockers to repurposed drugs and natural products, have shown promising antitumor effects in OSCC models. Nanomedicine approaches further expand therapeutic potential by ensuring tumor selectivity and minimizing systemic toxicity. Conversely, ferroptosis inhibitors may protect against tissue injury in inflammatory oral diseases, illustrating the context-dependent nature of ferroptotic modulation. The emergence of ferroptosis-related biomarkers, including gene signatures and non-coding RNAs, offers tools for prognostication and patient stratification, paving the way toward precision medicine. Notably, a prognostic model based on eight ferroptosis-related lncRNAs was recently developed and validated in OSCC, demonstrating independent predictive value for overall survival and highlighting the clinical utility of ferroptosis-associated transcriptomic signatures [154].

Looking forward, several challenges must be addressed before ferroptosis-based interventions can enter clinical practice. Safe and pharmacologically viable GPX4 inhibitors remain under development, and robust validation of ferroptosis biomarkers in prospective patient cohorts is still lacking. Optimizing multimodal regimens that integrate ferroptosis with chemotherapy, radiotherapy, or immunotherapy will be essential, while carefully balancing efficacy against potential toxicity in normal tissues and immune cells. In inflammatory oral diseases, further work is needed to define when ferroptosis should be inhibited to preserve tissue integrity and when its induction could benefit host defense.

If these translational hurdles can be overcome, ferroptosis has the potential to reshape therapeutic paradigms in oral oncology and beyond. By converting a fundamental cell death mechanism into a clinical tool, ferroptosis research offers not only a strategy to overcome persistent resistance in OSCC but also a framework to redefine the treatment of diverse oral diseases.

In conclusion, this review uniquely integrates molecular mechanisms, redox and lipid metabolic regulation, and translational therapeutic strategies targeting ferroptosis in OSCC. By bridging mechanistic discoveries with clinical prospects, it provides a novel conceptual framework for exploiting ferroptosis as a therapeutic vulnerability in oral cancer and related oral pathologies.

## Figures and Tables

**Table 1:** Key regulators of ferroptosis in OSCC.

Category	Molecule/ Pathway	Function in OSCC	Effect on Ferroptosis	Expression Pattern in Cell Line(S)	References
Antioxidant defenses	GPX4	Detoxifies lipid hydroperoxides	Inhibits ferroptosis	Upregulated (confers resistance)	[77]
	SLC7A11 (system Xc^−^)	Imports cystine for GSH synthesis	Inhibits ferroptosis	Upregulated	[25]
	SLC3A2 (system Xc^−^ partner)	Forms heterodimer with SLC7A11; regulates amino acid transport, immune evasion, and metastasis in OSCC	Inhibits ferroptosis	Downregulated in immune evasion	[78]
	FSP1	Regenerates CoQ_10_H_2_ via NADPH	Inhibits ferroptosis	Upregulated in drug-tolerant cells	[23,79]
	Nrf2 (NFE2L2)	Activates GPX4/SLC7A11, HO-1	Mostly inhibits ferroptosis	Upregulated/hyperactivated	[66,80]
Transcription factors	Nrf1 (NFE2L1) → HJURP	Binds HJURP promoter, upregulates GPX4/SLC7A11, reduces lipid ROS, promotes OSCC proliferation and resistance	Inhibits ferroptosis	Upregulated	[81]
Tumor suppressors	p53	Represses SLC7A11	Promotes ferroptosis	Mutated or inactivated (↓ functional)	[82]
	PER1 → HIF-1α	Reduces HIF-1α, ROS/LPO	Promotes ferroptosis	Downregulated	[24]
	TCF12	Represses OTUB1, destabilizes SLC7A11, restores cisplatin sensitivity	Promotes ferroptosis	Downregulated	[61]
Oncogenes	AEBP1	Activates JNK/p38/ERK	Inhibits ferroptosis	Upregulated	[83]
	PPT1	Stabilizes GPX4	Inhibits ferroptosis	Upregulated	[32]
	CK19	Suppresses ACSL4	Inhibits ferroptosis	Upregulated	[54]
	CDH4 (R-cadherin)	Upregulated in OSCC; enhances proliferation, migration, EMT; elevates GPX4 and GSH, reduces lipid peroxidation; poor survival correlation	Inhibits ferroptosis	Upregulated	[84]
Metabolic enzymes	ACSL4, LPCAT3	Incorporate PUFAs into membranes	Promote ferroptosis	Downregulated	[26]
	TPI1	Reduces ROS/LPO, cisplatin resistance	Inhibits ferroptosis	Upregulated	[31]
Epigenetic factors	circ_0000140/miR-527/SLC7A11	Upregulates SLC7A11, cisplatin resistance	Inhibits ferroptosis	Upregulated	[30]
	miR-26a-5p	Targets SLC7A11 3′UTR, decreases cystine uptake and GSH, sensitizes OSCC to ferroptosis	Promotes ferroptosis	Downregulated	[60]
	STARD4-AS1 (lncRNA)	Enhances proliferation, LPO	Inhibits ferroptosis	Upregulated	[29]
	METTL3 (m^6^A methyltransferase)	Stabilizes SLC7A11 mRNA via m^6^A modification, enhancing ferroptosis resistance	Inhibits ferroptosis	Upregulated	[85,86]
	FTO (m^6^A demethylase)	Destabilizes ACSL3 and GPX4 mRNA via m6A demethylation, enhancing ferroptosis	Promotes ferroptosis	Downregulated	[87]
Protein quality control	HSPA5 (GRP78)	ER chaperone; its downregulation accompanies ferroptosis in OTSCC	Inhibits ferroptosis (loss → ↑ferroptosis)	Downregulated during RSL3 or erastin treatment	[34]
Immune checkpoint	PD-L1 (intrinsic function)	Activates SOD2, maintains redox homeostasis; loss increases ferroptosis and immunogenic death	Inhibits ferroptosis	Upregulated	[88]
TME-derived regulators	PDPN^+^ CAF–exosomal lncRNA FTX	Transfers to OSCC cells, forms FTX/FEN1 complex, suppresses ACSL4-mediated ferroptosis, enhances motility and invasiveness	Inhibits ferroptosis	Upregulated in CAF-rich OSCC	[89]
ER stress regulators	REEP6	Maintains ER homeostasis, downregulates ACSL4, confers resistance to RSL3	Inhibits ferroptosis	Upregulated	[90]
Transcriptional co-activators	YAP/TAZ–ACSL4 axis	Activated by manganese; phase separation promotes ACSL4 activation; correlates with survival	Promotes ferroptosis	Upregulated under Mn exposure	[53]

Abbreviations: ACSL4, acyl-CoA synthetase long-chain family member 4; AEBP1, adipocyte enhancer-binding protein 1; ADCD, autophagy-dependent cell death; CAF, cancer-associated fibroblast; CDH4, cadherin-4; CK19, cytokeratin-19; ER, endoplasmic reticulum; FSP1, ferroptosis suppressor protein 1; GPX4, glutathione peroxidase 4; GSH, glutathione; HIF-1α, hypoxia-inducible factor 1α; HJURP, Holliday junction recognition protein; HSPA5, heat shock protein family A member 5; HO-1, heme oxygenase-1; lncRNA, long non-coding RNA; LPCAT3, lysophosphatidylcholine acyltransferase 3; METTL3, methyltransferase-like 3; Nrf2, nuclear factor erythroid 2–related factor 2; OSCC, oral squamous cell carcinoma; PD-L1, programmed death-ligand 1; PDPN, podoplanin; PER1, period circadian regulator 1; PUFA, polyunsaturated fatty acid; REEP6, receptor expression–enhancing protein 6; ROS, reactive oxygen species; SLC7A11, solute carrier family 7 member 11; STARD4-AS1, steroidogenic acute regulatory-related lipid transfer domain protein 4 antisense RNA 1; TME, tumor microenvironment; YAP, Yes-associated protein; TAZ, transcriptional co-activator with PDZ-binding motif.

**Table 2:** Therapeutic strategies targeting ferroptosis in OSCC.

Strategy	Agent/Example	Mechanism	Preclinical OSCC Evidence	References
System Xc^−^ inhibitors	Erastin, sulfasalazine	Block cystine uptake → ↓GSH/GPX4	Induce ferroptosis, restore cisplatin sensitivity	[97,119]
GPX4 inhibitors	RSL3, FIN56	Direct GPX4 inhibition → ↑LPO	Ferroptotic death in CAL27, SCC9, HSC3	[87]
Iron modulators	Ferritinophagy activators	↑Labile Fe^2+^, lipid ROS	Enhance ferroptosis	[45]
Repurposed drugs	Artesunate	Iron-dependent ROS, ↑LIP	Induces ferroptosis, synergy with cisplatin	[32,66]
	Sorafenib	System Xc^−^ inhibitor	Enhances cisplatin sensitivity	[120]
	Trifluoperazine (TFP)	GPX4 inhibition, autophagy	Induces ferroptosis, poor prognosis with GPX4^high^	[108]
	Quisinostat (HDACi)	ROS stress, lipid peroxidation	Sensitizes OSCC to ferroptosis	[121]
	Disulfiram (±Cu)	Nrf2/HO-1 modulation	Promotes ferroptosis in OSCC	[67]
	Melatonin	ROS amplification, mitochondrial stress	Potentiates erastin-induced ferroptosis	[122]
Natural compounds	Piperlongumine	↓GPX4/SLC7A11, ↑ROS	Suppresses growth, synergizes with CB-839	[33]
	Evodia lepta extract	↓GPX4/HSPA5, ↓PD-L1	Cytotoxic and immunomodulatory	[34]
	Quercetin	Inactivates mTOR/S6K	Induces ferroptosis, enhances cisplatin	[107]
	Brusatol	Inhibits Nrf2/GCLC, ↓SLC7A11, GSH depletion	Promotes ferroptosis, suppresses OSCC growth in vitro and in vivo	[123]
	Resveratrol	Activates p53, represses SLC7A11, ↓GSH, ↑Fe^2+^/ROS	Induces ferroptosis, inhibits OSCC proliferation and invasion	[82]
	Fucoxanthin	Downregulates Nrf2/HO-1/GPX4, ↑ROS, ↑Fe^2+^, ↑p53	Induces ferroptosis in SCC-25 tongue carcinoma cells	[124]
	Baicalin	Suppresses FTH1, ↓EMT, ↑ferroptosis	Inhibits proliferation and invasion in OSCC cells	[125]
	Ganoderma lucidum spore powder (A-GSP)	↑Fe^2+^ influx, GSH depletion, ↑ACSL4, ↓GPX4; induces mitochondrial dysfunction	Induces ferroptosis, suppresses OSCC growth in vivo	[126]
Nanomedicine	MnO_2_-RSL3 NPs	ROS amplification, GSH depletion	“Explosive” ferroptosis in vivo	[109]
	Carbon dot–hydrogel films	Fe^3+^ detection + ferroptosis	Theranostic system in OSCC	[110]
	Fe-dopamine composites	Fenton-like ROS generation	Induce lipid peroxidation	[127]
	Sorafenib–Ce6 nanoparticles	Photodynamic + ferroptosis	Overcome hypoxia resistance	[120]
	Exo-AuMn nanoclusters	ROS generation, immune targeting	Selective ferroptosis + imaging	[128]
	CD44-targeted mP6/Rg3 micelles	Inhibit ABCB1, promote ferroptosis in cancer stem cells	Suppress CSC proliferation, migration, and OSCC growth in vitro and in vivo	[129]
Combination therapies	Cisplatin + RSL3/erastin	Chemo-ferroptosis synergy	Overcome cisplatin resistance	[26,30]
	*Evodia lepta* + cisplatin	GPX4/HSPA5 suppression + cytotoxicity	Enhance chemosensitivity	[34]
	Carnosic acid + cisplatin	Inactivation of Nrf2/HO-1	Reverse cisplatin resistance	[102]
	Amoxicillin + cisplatin	Mitochondrial dysfunction, ferroptosis	Enhance cisplatin efficacy	[130]
	RSL3 + LYN-1604	Autophagy + ferroptosis induction	Synergistic tumor suppression	[36]
	Radiotherapy + ferroptosis inducers	Radiation-induced ROS + ferroptosis	Radiosensitization in OSCC	[113,114]
	Hyperbaric oxygen + ionizing radiation	Suppresses GPX4, enhances ferroptosis	Re-sensitizes radio-resistant OSCC cells, improves tumor control	[131]
	Photodynamic therapy: Ce6–erastin nanodrug	Relieves hypoxia, inhibits SLC7A11, sustained ROS via Fenton reaction	Enhances PDT efficacy in CAL-27 and xenograft models	[132]
	Astaxanthin + ionizing radiation	Inhibits GPX4/SLC7A11, ↑ACSL4, ↑ferroptosis	Enhances radiosensitivity in OSCC cells and xenografts	[117]
	Immunotherapy + ferroptosis	PD-L1 downregulation, immune activation	Enhance ICI efficacy	[34,133]

**Table 3:** Clinical implications and future directions of ferroptosis in oral diseases.

Domain	Current Evidence	Potential Clinical Impact	Future Directions	References
OSCC prognosis	FRG signatures; circRNAs/lncRNAs (e.g., circ_0000140, STARD4-AS1); 8 ferroptosis-related lncRNAs model; FGS validated in HNSCC/OSCC, linked to CD276^+^ fibroblasts and ATG5-mediated immune exclusion	Biomarker-driven risk stratification; prediction of immunotherapy responsiveness	Large prospective validation	[76,92,142,154]
Bioinformatics/systems biology	Network pharmacology identified GSH-related ferroptosis targets (EGFR, PTGS2, HIF1A, SLC3A2, etc.)	Provides candidate targets for therapy and biomarkers	Integrate in silico predictions with experimental validation	[141]
Chemoresistance	Ferroptosis suppressed in cisplatin-resistant OSCC; FSP1 upregulated in drug-tolerant persister cells	Restore cisplatin sensitivity via FINs, ncRNA targeting, FSP1 inhibition in persister cells	Clinical trials combining cisplatin + FINs	[26,30,79]
Radiotherapy	Radiation induces lipid ROS, ferroptosis enhances radiosensitivity	Radiosensitization	Nanoparticle-mediated ROS amplification	[113,114]
Immunotherapy	Ferroptosis boosts immunogenicity, but may impair T cells	Guide ICI combinations	Balance tumor vs. immune ferroptosis	[34,93]
Non-malignant diseases	Pulpitis, periodontitis linked to ferroptosis-driven injury	Ferroptosis inhibitors may protect tissues	Disease-specific studies	[6,148]

## Data Availability

No new data were created or analyzed in this study.

## Abbreviations

ACSL4: acyl-CoA synthetase long-chain family member 4; ADCD, autophagy-dependent cell death; AEBP1, adipocyte enhancer-binding protein 1; CAF, cancer-associated fibroblast; CDH4, cadherin-4; CK19, cytokeratin 19; CoQ_10_, coenzyme Q_10_; CSC, cancer stem cell; DAMPs, damage-associated molecular patterns; DDP, cisplatin; DHODH, dihydroorotate dehydrogenase; DRP1, dynamin-related protein 1; EMT, epithelial–mesenchymal transition; ER, endoplasmic reticulum; FEN1, flap endonuclease 1; FIN, ferroptosis inducer; FRG, ferroptosis-related gene; FSP1, ferroptosis suppressor protein 1; GPX4, glutathione peroxidase 4; GSH, glutathione; HDAC, histone deacetylase; HJURP, Holliday junction recognition protein; HO-1, heme oxygenase-1; HNSCC, head and neck squamous cell carcinoma; ICI, immune checkpoint inhibitor; LIP, labile iron pool; LPCAT3, lysophosphatidylcholine acyltransferase 3; lncRNA, long non-coding RNA; MALAT1, metastasis-associated lung adenocarcinoma transcript 1; MDA, malondialdehyde; MDSC, myeloid-derived suppressor cells; mTOR, mechanistic target of rapamycin; NAC, *N*-acetylcysteine; ncRNA, non-coding RNA; NFE2L1, nuclear factor erythroid 2-like 1; NFE2L2 (Nrf2), nuclear factor erythroid 2-related factor 2; OSCC, oral squamous cell carcinoma; PDPN, podoplanin; PER1, period circadian regulator 1; PE, phosphatidylethanolamine; PL, phospholipid; PPT1, palmitoyl-protein thioesterase 1; PUFA, polyunsaturated fatty acid; RCD, regulated cell death; ROS, reactive oxygen species; RSL3, RAS-selective lethal small molecule 3, a GPX4 inhibitor; SLC3A2, solute carrier family 3 member 2; SLC7A11, solute carrier family 7 member 11; TCF12, transcription factor 12; TFP, trifluoperazine; TfR1, transferrin receptor 1; TME, tumor microenvironment; TPI1, triosephosphate isomerase 1; Xc^−^, cystine/glutamate antiporter system.
